# Idiopathic Central Precocious Puberty Associated with 11 Mb *De Novo* Distal Deletion of the Chromosome 9 Short Arm

**DOI:** 10.1155/2013/978087

**Published:** 2013-07-31

**Authors:** Mariangela Cisternino, Erika Della Mina, Laura Losa, Alexandra Madè, Giulia Rossetti, Lorenzo Andrea Bassi, Giovanni Pieri, Baran Bayindir, Jole Messa, Orsetta Zuffardi, Roberto Ciccone

**Affiliations:** ^1^Department of Pediatrics, IRCCS Policlinico San Matteo, University of Pavia, Piazzale Golgi 19, 27100 Pavia, Italy; ^2^Department of Molecular Medicine, University of Pavia, Via Forlanini 6, 27100 Pavia, Italy; ^3^IRCCS C. Mondino National Neurological Institute Foundation, Via Mondino 2, 27100 Pavia, Italy

## Abstract

We report a girl with a *de novo* distal deletion of 9p affected by idiopathic central precocious puberty and intellectual disability. Genome-wide array-CGH revealed a terminal deletion of about 11 Mb, allowing to define her karyotype as 46; XX, del(9)(p23-pter). To our knowledge, this is the second reported case of precocious puberty associated with 9p distal deletion. A third case associates precocious puberty with a more proximal 9p deletion del(9)(p12p13,3). In our case, more than 40 genes were encompassed in the deleted region, among which, DMRT1 which is gonad-specific and has a sexually dimorphic expression pattern and ERMP1 which is required in rats for the organization of somatic cells and oocytes into discrete follicular structures. Although we cannot exclude that precocious puberty in our del(9p) patient is a coincidental finding, the report of the other two patients with 9p deletions and precocious puberty indeed suggests a causative relationship.

## 1. Introduction

Central precocious puberty (CPP) is classically defined by the appearance of sexual secondary characteristics before the age of 8 years in girls and 9 years in boys [1]. It is caused by a premature activation of the hypothalamus-pituitary-gonadal axis. CPP may be either idiopathic or associated with occult intracranial lesion, mainly hypothalamic hamartoma or astrocytoma and noncancerous CNS disorders [2, 3]. This condition may cause early epiphyseal maturation with compromised final height as well as psychological stress [4, 5].

 Chromosome 9p deletion syndrome (OMIM#158170) is a well-recognized entity, caused by a constitutional monosomy of a portion of 9p of different sizes in different patients. It was first described by Alfi et al. in 1973 [6]. Until now, approximately 180 cases have been published [7]. 

The most common features of monosomy 9p syndrome, as described by Swinkels et al. [8], include developmental and psychomotor delay, trigonocephaly, flat midface, short palpebral fissures, highly arched eyebrows, low-set ears, short flat nose with anteverted nostrils, thin upper lip, long philtrum, high palate, micrognathia, short neck, nipple hypertelorism, tapering fingers, flat feet, hypotonia, and developmental sex disorders in XY subjects. The critical region for a consensus phenotype has been reported to be located in a 300 Kb region on 9p22.3 [8]. Approximately half of the cases are due to *de novo* deletions of 9p, the remaining ones to unbalanced translocations with a derivative 9p chromosome. Few cases have been reported with 9p distal deletion concomitant to 9q distal duplication, with some of them due to a parental chromosome 9 inversion.

To our knowledge, only one case of distal 9p deletion [del9(p22pter)] [9] and a second case of proximal 9p deletion [del9(p12p13.3)] [10] have been reported in association with precocious puberty.

Here, we report a girl with distal 9p deletion with idiopathic central precocious puberty and mental impairment.

## 2. Case Presentation

The proband is a girl. We have been following her from the age of 7 years and 4 months. She is the only child of nonconsanguineous healthy Italian parents. At her birth, her mother was 29 years old, and her father was 31 years old. She was born at the 40th week of gestation after uneventful pregnancy and delivery. Her birth weight was 3350 g (50th centile), her length was 50 cm (50th centile), and her head circumference was 35 cm (75th centile). She had neonatal jaundice requiring one-day phototherapy. Her psychomotor development was delayed: she began to walk by the age of 24 months. She suffered from chronic constipation for 2 and a half years. At the age of 6 years, she presented a left inguinal hernia which was surgically reduced.

She has a moderate intellectual disability with good social adaptation, and presently, she is enrolled in a public school with the aid of a tutor.

**Figure 1:**
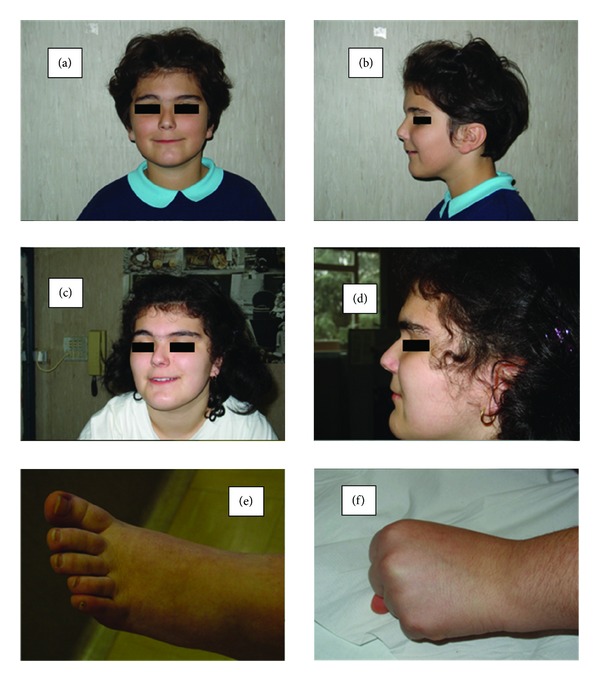
Pictures of the girl affected by 9p-syndrome. (a), (b): face and profile of the patient at the age of 7 years. (c), (d): face and profile of the patient at the age of 14 years. (e), (f): shortness of 4th metacarpal and metatarsal.

At 7 years and 4 months of age, she was referred to our unit of Pediatric Endocrinology for evaluation of precocious puberty since pubarche, axillarche, and axillary sweating were noted at the age of 7 years, followed by unilateral thelarche and pubertal spurt. Clinical evaluation showed several facial anomalies (Figure 1), including low anterior hair line, low-set ears, synophrys, short nose, long philtrum, and wide mouth with thin vermilions of the lower and upper lip. In addition, geographic tongue, dental crowding, slightly arched palate, bilateral short 4th and 5th metacarpal and metatarsal, and signs of ungueal decalcification were noted. She was 128 cm tall (H-SDS +1.19), and her weight was 28.3 kg (90th percentile) with a BMI of 17.3 (BMI-SDS +1.35). Pubertal stage (according to Marshall and Tanner) [12] was unilateral B2, PH2, and A2. Bone age was of 10 years according to the Greulich and Pyle atlas [13]. Predictable adult height (according to the Bayley & Pinneau method) was of 154.5 cm (target height 160.2 cm).

Hormonal investigations showed basal luteinizing hormone (LH) of 0.1 IU/L and basal follicle-stimulating hormone (FSH) of 5.4 IU/L (normal prepubertal values: LH < 0.5 IU/L and FSH < 7 IU/L), and the serum estradiol (E2) level was slightly increased 26.2 pg/mL (normal prepubertal values < 15 pg/mL). The GnRH stimulation test showed an LH and FSH response consistent with a prepubertal stage (LH peak 3.7 IU/L, FSH peak 20.7 IU/L; normal prepubertal values: LH < 7 IU/L, FSH 18.2 ± 2.62 IU/L). Measurement of adrenal androgen levels showed dehydroepiandrosterone sulphate (DHEA-S) values of 0.6 mcg/mL which were consistent with adrenarche. Pelvic ultrasonography (US) showed no masses, and ovarian and uterine volumes were not enlarged and were normal for chronological age. Premature adrenarche was then diagnosed; however, because of the bone age advancement and the increased E2 levels, the girl was subsequently re-examinated.

Eight months later, at the age of 8 years, the patient showed a progression of thelarche, which became bilateral, and of pubarche, whereas the growth rate remained higher than normal; the GnRH stimulation test showed a pubertal LH response (peak 11.8 IU/L), and the pelvic US showed increased size of the uterus (6 cm^3^) and of the ovaries (2 cm^3^, bilaterally). The uterus was pear-shaped, and the ratio between the fundal and cervical anteroposterior diameters was >1. The girl was then diagnosed as a central precocious puberty. Brain MRI did not show any abnormality, including in the hypothalamic and pituitary regions; therefore, the origin of the precocious puberty was considered idiopathic. A suppressive treatment of puberty with a GnRH analogue (3.75 mg per month of a depot suspension of leuprolide acetate) was administered until the age of 12 years. The menarche appeared at the age of 13.1 years (final height 161.5 cm, H-SDS −0.11), followed by irregular menses and polymenorrhea which required periodical iron supplementation. At the age of 14 years, she complained of a unilateral hypertrophy of the labia minora causing local pain, irritation, and psychosocial distress, which required labiaplasty. 

Additional investigations, during years, showed normal hepatic, renal, and thyroid function; hormonal profile was normal except for elevated levels of prolactin (max. 57.4 ng/mL) which was probably functional as no hypophyseal adenoma was found with MRI. No treatment for hyperprolactinemia was necessary due to the lack of specific symptoms, that is, galactorrhea and amenorrhea.

EEG showed focal paroxysms in the right hemisphere, not requiring antiepileptic therapy. 

## 3. Cytogenetics and Array-CGH

Standard karyotype revealed a normal female karyotype.

A terminal deletion of the short arm of chromosome 9 [46, XX.ish.del(9p) (pVYS234B-9)] was detected by subtelomeric fluorescence *in situ* hybridization (FISH) analysis (Vysis kit).

Conventional cytogenetics and FISH analysis of patient's parents demonstrated that the deletion occurred *de novo*.

Genome-wide array-CGH has been performed using the Human Genome CGH Microarray Kit 4x44K (Agilent Technologies, Santa Clara, CA, USA) according to the manufacturers' protocol [14]. The investigation was made using a female genomic DNA pool (Promega Ltd.) as reference, and results has been referred as UCSC hg18 (NCBI Build 36), March 2006.

**Figure 2:**
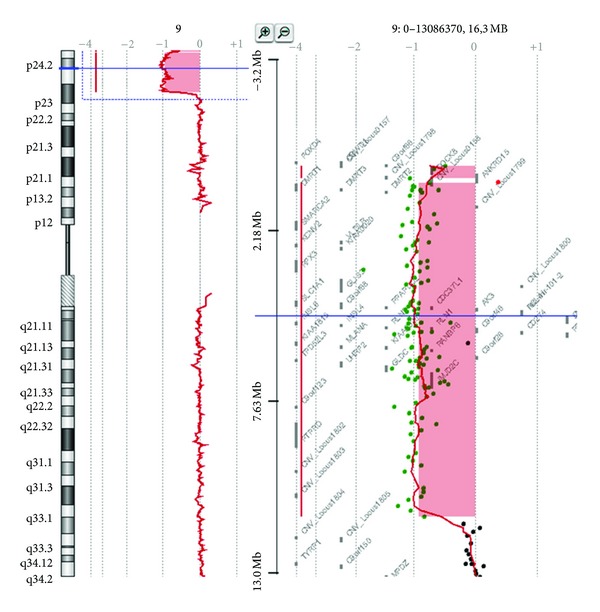
Array-CGH profile of the patient showing the whole chromosome 9 (left) and an enlargement of the short arm with the 11 Mb deletion at 9p23-pter 8 (right).

This analysis revealed a terminal deletion of about 11 Mb in the short arm of chromosome 9 [del(9)(p23-pter)]. Proximal breakpoint is between 11,259549 Mb (last deleted) and 11,447340 Mb (first not deleted); the last oligomer present on the platform was localized at 204,367 Kb (Figure 2).

## 4. Discussion

We present a case of distal 9p deletion which came to our attention because of idiopathic central precocious puberty.

About 180 cases of monosomy 9p syndrome have already been described [7]. The cases that presented del(9p) as the sole anomaly usually have a *de novo* mutation [15], which was the case of our patient. In the majority of cases, the breakpoint occurs at 9p21 [16]; however, many clinical features are similar regardless of the length of the deletion [15].

Even if our patient is different from previous del(9p) cases in the absence of trigonocephaly, flat nasal bridge, and the large number of digital whorls, she has many features in common with classic 9p-syndrome such as developmental delay and moderate mental retardation, long philtrum, arched palate, low-set ears, low anterior hair line, cardiac defect, and inguinal hernia. Besides, she has some physical characteristics that are not classical features of 9p-syndrome such as short 4th metacarpal and metatarsal and geographic tongue (Table 1).

However, what we consider to be important in this case is the fact that our patient was affected by central precocious puberty, from the age of 7 years, requiring suppressive therapy. To our knowledge, this is the second reported case of precocious puberty associated to 9p distal deletion. The first case was a boy described in 1979 by Funderburk et al. [9] who carried a *de novo* [del9(p22->pter)]. The unusual features of the boy were precocious puberty from the age of 8 years and 10 months and hexadactily. Precocious puberty was not mentioned in other cases with distal 9p deletion that should have already undergone pubertal development according to their age. Several del(9p) patients had anomalous external genitalia such as hypoplastic labia majora and prominent labia minora [9], as was observed in this case. 

In our patient, more than 40 genes were encompassed in the deleted region. Some of these genes are involved in an insulin metabolism pathway. Increased insulin and IGF1 levels were found elevated in girls with CPP, suggesting a causal interrelation between CPP and insulin secretion [17, 18]. Unfortunately, the insulin secretion was not studied at the time of the onset of CPP in our patient. The rearrangement included also DMRT1: this gene is found in a cluster with two other members of the gene family, having in common a zinc finger-like DNA-binding motif (DM domain). This gene exhibits a gonad-specific and sexually dimorphic expression pattern. Defective testicular development and XY feminization occur when this gene is hemizygous. Recent studies on its function revealed that DMRT1 protein controls Stra8 specifically, activating it in the fetal mouse ovary [19].

A gene that could be interesting in relation to the precocious puberty present in our del(9p) patient is ERMP1 (fxna rat homolog of KIAA1815). The protein product of this gene, in a rat, is required for the organization of somatic cells and oocytes into discrete follicular structures. No ERMP1 mutations have been reported in humans [20].

By differential display in the neonatal rat ovary, Garcia-Rudaz et al. [20] identified a novel cDNA, termed fxna (felix-ina), expressed during folliculogenesis.

Obviously, we cannot exclude that precocious puberty in our del(9p) patient is a coincidental finding; although, the report of other two patients with 9p deletions and precocious puberty (a male with a similar deletion and a female with a cytogenetically identified more proximal 9p deletion) indeed suggests a causative relationship. 

The association of moderate mental retardation and CPP has been described in patients carrying several genetic anomalies, other than the 9p distal deletion, detected with the CGH array technique and the fluorescence in situ hybridization analysis (FISH) [21–24]. The molecular basis of this association remains unknown, but it is likely that multiple gene aberrations are responsible for this association.

## Figures and Tables

**Table 1:** Clinical features of 9p-syndrome, as described in OMIM web site [11], compared to the clinical features presented by our patient affected of 9p-syndrome due to 9p24.3-p23 deletion.

Category	Features	Present case
Head	Trigonocephaly	No

Face	Midface hypoplasia	No
	Long philtrum	Yes
	Micrognathia	No

Ears	Low-set ears	Yes
	Malformed ears	No
	Posteriorly angulated ears	Yes

Eyes	Upslanting palpebral fissures	Yes
	Hypertelorism	Yes
	Epicanthal folds	Yes
	Small palpebral fissures	Yes
	Myopia	No
	High-arched eyebrows	Yes

Nose	Flat nasal bridge	Yes
	Anteverted nares	No
	Choanal atresia	No

Mouth	Thin upper lip	Yes
	Microstomia	No
	High narrow palate	Yes

Neck	Short neck	No

Heart	Heart murmurs	Yes
	Congenital cardiac malformations	No
	Atrial septal defect	No
	Ventricular septal defect	No
	Patent ductus arteriosus	No

Breasts	Widely spaced nipples	No

Abdomen	Inguinal hernia	Yes
	Omphalocele	No

Skeletal	Scoliosis	No
	Tapering fingers	No
	Pes planus	No

Skin, nails, and hair	Pale skin	Yes
	Hyperconvex nails	No
	High arched eyebrows	No

CNS	Mental retardation	Yes
	Delayed psychomotor development	Yes
	Speech delay	Yes
	Hypotonia	Yes

		Central precocious puberty

		Short metacarpal
		Short metatarsal

		Wide mouth
		Dental crowding
		Geographic tongue

		Ungueal decalcification

		Low anterior hairline
		Synophrys
